# Metaheuristic integrated machine learning classification of colon cancer using STFT LASSO and EHO feature extraction from microarray gene expressions

**DOI:** 10.1038/s41598-024-67135-1

**Published:** 2024-07-17

**Authors:** Ajin R. Nair, Harikumar Rajaguru, M. S. Karthika, C. Keerthivasan

**Affiliations:** 1 Department of Electronics and Communication Engineering, Bannari Amman Institute of Technology, Sathyamangalam, India; 2 Department of Information Technology, Bannari Amman Institute of Technology, Sathyamangalam, India; 3 Bannari Amman Institute of Technology, Sathyamangalam, India; 4 Procyon TechSolutions Private Limited, Bengaluru, India

**Keywords:** STFT, LASSO, EHO, Lung cancer, Microarray gene expression, GMM, PSO GMM, DFA, NBC, Firefly GMM, SVM, Flower pollination optimization with GMM, Cancer, Biomarkers, Diseases, Health care, Medical research, Engineering

## Abstract

The microarray gene expression data poses a tremendous challenge due to their curse of dimensionality problem. The sheer volume of features far surpasses available samples, leading to overfitting and reduced classification accuracy. Thus the dimensionality of microarray gene expression data must be reduced with efficient feature extraction methods to reduce the volume of data and extract meaningful information to enhance the classification accuracy and interpretability. In this research, we discover the uniqueness of applying STFT (Short Term Fourier Transform), LASSO (Least Absolute Shrinkage and Selection Operator), and EHO (Elephant Herding Optimisation) for extracting significant features from lung cancer and reducing the dimensionality of the microarray gene expression database. The classification of lung cancer is performed using the following classifiers: Gaussian Mixture Model (GMM), Particle Swarm Optimization (PSO) with GMM, Detrended Fluctuation Analysis (DFA), Naive Bayes classifier (NBC), Firefly with GMM, Support Vector Machine with Radial Basis Kernel (SVM-RBF) and Flower Pollination Optimization (FPO) with GMM. The EHO feature extraction with the FPO-GMM classifier attained the highest accuracy in the range of 96.77, with an F1 score of 97.5, MCC of 0.92 and Kappa of 0.92. The reported results underline the significance of utilizing STFT, LASSO, and EHO for feature extraction in reducing the dimensionality of microarray gene expression data. These methodologies also help in improved and early diagnosis of lung cancer with enhanced classification accuracy and interpretability.

## Introduction

Colon cancer stands as a formidable public health adversary, claiming countless lives annually due to its late detection and devastating potential as mentioned in Jemal et al.^1^. Early identification maximizes treatment success and offers a beacon of hope, significantly improving treatment efficacy and long-term prognosis for patients as discussed in Veer et al.^2^. There are several medical methods to diagnose Colon cancer, which are mentioned in the research and literature. The Stool Occult Blood Test (FOBT) is a simple test that checks stool samples for hidden blood used to detect colon cancer. The hidden blood can be a sign of colon cancer or other conditions. However, the FOBT is a non-invasive and convenient test that recognises colon cancer in advanced stages as discussed in Merak et al.^3^. A biopsy is a method that removes a small tissue sample from a suspicious colon area to visually examine and diagnose cancer under a microscope by a pathologist. As indicated in Compton et al.^4^, Biopsy turns out to be an invasive method involving bleeding risks, but can confirm cancer along with the type and grade of cancer stage. Miller et al.^5^ discuss about Barium Enema (BaEn), a different method that captures X-ray images of the colon area. The colon area will be filled with barium, a contrast material for X-ray image visualization. BaEn involves radiation exposure and can reveal polyps, masses, or the narrowing of the colon part. It offers a broader view of the colon compared to FOBT, but may not detect small polyps as indicated by Ott et al.^6^.

Microarray gene expression analysis examines the activity of thousands of genes simultaneously. It offers several advantages over traditional clinical methods for diagnosing colon cancer. Firstly, as pointed out in Poturnajova et al.^7^, it provides a comprehensive molecular profile of the tumour, allowing for the identification of specific gene expression patterns associated with cancer development and progression. As given in Zhang et al.^8^, compared to ultrasonic scanning, molecular information provided by microarray gene expression analysis offers distinct advantages in diagnosing colon cancer. The identification of specific genetic alterations associated with colon cancer development and progression is thus possible. The molecular information offers insights into personalized treatment strategies and prognosis, surpassing the capabilities of ultrasound in providing detailed molecular insights into the disease process as described in Vaidya et al.^9^.

Moreover, microarray analysis can distinguish between different subtypes of colon cancer based on their gene expression profiles as indicated in Bertucci et al.^10^, which may have prognostic implications and influence treatment decisions. Xavier et al.^11^ details that Microarray gene expression also helps to identify new biomarkers for colon cancer diagnosis, leading to the development of more specific and sensitive tests. Furthermore, microarray gene expression analysis can be performed using minimally invasive techniques, such as obtaining tumour tissue samples via biopsy or analysing circulating tumour cells or cell-free DNA in the blood, reducing patient discomfort and risk compared to invasive procedures like colonoscopy as discussed in Galamb et al.^12^. Overall, microarray gene expression analysis offers a more precise, sensitive, and minimally invasive approach to diagnosing colon cancer, potentially leading to earlier detection, more accurate prognosis, and tailored treatment strategies for patients. However, as marked in Wulfkuhle et al.^13^, microarray technology is still under development for routine clinical use and requires complex analysis and further validation before widespread adoption. The vast amount of data generated by microarrays poses a major challenge known as the "curse of dimensionality," as sermonized in Maniruzzaman et al.^14^, necessitating dimensionality reduction techniques and feature extraction techniques for effective classification and analysis of microarray data as sermonized in Guyon et al.^15^. So, in this research, we focus on leveraging the dimensionality of microarray gene expression data and select relevant features that aid in improving the classification accuracy. The various dimensionality reduction and feature extraction methods with be discussed in the upcoming section.

### Review of previous work

There are various research works performed in the literature based on dimensionality reduction and feature extraction methods on microarray gene data for various cancer databases. The selection of methodology to perform dimensionality reduction and feature extraction is significant in reducing the data volume and extracting relevant features and patterns. This selection aids the upcoming classification phase to improve the classification accuracy and reduce the overfitting problems. The diverse feature extraction/dimensionality techniques with classification, pronounced in the literature are provided in Table 1.

**Table 1:** Review of previous work.

Sl.No.	Author and year	Database	Feature extraction/dimensionality reduction technique	Classifiers used	Evaluation metrics
1	Liu et al. (2013)^16^	Colorectal cancer Dataset (Prof.Lindy Durant’s research)	Continuous Wavelet Transform (CWT)	Genetic Algorithm based on Bayes classifier	Accuracy = 78.6%
2	Islam et al. (2023)^17^	Ischemic sensitivity dataset	genomap	genomap + genoNet	Accuracy = 93%
3	Xiao et al. (2018)^18^	TCGA Database	Stacked sparse auto-encoder (SSAE)	Support Vector Machine (SVM), Neural Network (NN), Random Forest (RF)	Accuracy = 99.89%
4	You et.al. (2013)^19^	(Bio) Dataset	F-test & Partial Least Squares (PLS), ReliefF and PLS, Recursive Feature Elimination and PLS	LDA,SVM, NBC,KNN (K-Nearest Neighbourhood), INN	96.43%
5	Bonev et al. (2008)^20^	Datasets from Broad Institute, Stanford Genomic Resources, and Princeton University	Mutual Information	KNN, SVM	Accuracy = 89%
6	Xu et al. (2018)^21^	Simulated Genome Dataset Available in Bioinformatics online	Extreme phenotype sampling (EPS)	LASSO	Accuracy = 90%
7	Torkey et al. (2021)^22^	METABRIC, Nature 2012, and Nat Commun 2016, COX-PASnet, and TCGA	Principal Component Analysis (PCA) with Sparse Autoencoders	Cox Regression, Random Survival Forest (RSF)	Accuracy = 98%
8	Abdulla et al. (2020)^23^	Leukemia and DLBCL dataset	Genetic Algorithm (GA) and Binary Gray Wolf Optimization (BGWO)	Random Forest (RF) and KNN	Accuracy = 95%
9	Li et al. (2018)^24^	Colon cancer and Leukemia dataset	Variants of LDA, LASSO Spectral regression discriminant analysis (SRDA), locality preserving projections (LPP), kernel discriminant analysis with spectral graph analysis (SRKDA)	LDA, RLDA, HLDA,NDA, SRDA, LPP, SRKDA, and Lasso SRDA	Accuracy = 84%
10	Zhang et al. (2015)^25^	Leukemia-ALLAML, SRBCT	Projection Matrix	kNN, Locally Linear Discriminant Embedding (LLDE) , DNE (Discriminant Neighbourhood Embedding)	Accuracy = 90%
11	Subhajit et al. (2015)^26^	SRBCT, ALL_AML and MLL microarray datasets	Particle Swarm Optimization (PSO)—adaptive KNN	SVM (Linear, RBF, Polynomial, Quadratic)	Accuracy = 95%
12	Nursabillilah et al. (2022)^27^	Breast Cancer Gene Expression Database	Information Gain, Relief and Fisher Score (filter-based methods), LASSO (embedded-based method), GA, PSO, harmony search, ant colony, artificial bee colony, firefly algorithm, cuckoo search, gravitational search, grey wolf, whale optimization	RF, KNN, Naïve Bayes (NB), Logistic Regression (LR), Fuzzy Logic, Artificial NN (ANN)	Accuracy = 90% to 100%
13	Wang et al. (2003)^28^	Leukemia, Colon Cancer, Brain tumours and NCI60	Fuzzy c-means clustering, Weighted/Mean component plane	Fisher's linear discriminant	Accuracy = 95%
14	Aziz et al. (2016)^29^	colon cancer, acute leukemia, prostate cancer, lung cancer II, and high-grade glioma	Independent component analysis (ICA) and fuzzy backward feature elimination (FBFE)	SVM and NB	Accuracy = 90%
15	Yaqoob et al. (2024)^30^	Breast cancer dataset (Kent Ridge)	Sine Cosine and Cuckoo Search Algorithm	SVM	Accuracy = 99%
16	Joshi et al. (2024)^31^	Colon cancer	Cuckoo Search and Spider Monkey Optimization	SVM and NB	Accuracy = 92%

Here are certain limitations and research directions observed in the diverse methodologies listed in Table 1. In Liu et al.^16^, low accuracy is obtained to more number of redundant features. Islam et al.^17^ and Xiao et al.^18^ reported high accuracies due to the applied deep learning methods. However, deep learning methods incurs high computational complexity. The proposed methods by You et al.^19^ are not suitable for datasets that are highly nonlinear in nature. Bonev et al.^20^ and Xu et al.^21^ showcased efficient feature extraction techniques but there is only limited exploration of diverse classifiers. Torkey et al.^22^ performed survival tests instead of classification and obtained good accuracies in the range of 98%. Abdulla et al.^23^ have proposed a cost sensitive feature selection method with accuracy in the range of 95%. Li et al.^24^ explored a variety of regression techniques, and reported 84% of accuracy due to the minimum search space exploration due to LASSO and LDA regressions. Zhang et al.^25^ achieved an accuracy of 90% but the methodology is sensitive to outliers and missing of sensitive information while handling nonlinear data. Subhajit et al.^26^ and Nursabillilah et al.^27^ attained an accuracy of 95%, but slight traces of overfitting are reported by the adopted metaheuristic methods. Wang et al.^28^ and Aziz et al.^29^ proposed methods that reported accuracies over 90%, but the methodologies adopted in these research works are not suitable for highly nonlinear datasets. Yaqoob et al.^30^ proposed Sine Cosine and Cuckoo Search Algorithm for feature selection and classification of Breast cancer. Low number of prominent genes (30) is selected to attain a classification accuracy of 99%. For colon cancer classification, Joshi et al.^31^ utilized Cuckoo Search and Spider Monkey Optimization as feature selection. The reported accuracy of the research consisting of 2000 genes is only 92%. Along with these benchmark literature works, the research also follows some of the insights provided by Arowolo et al.^32^ that investigate various feature Extraction methods to classify Colon Cancer from microarray Data.

Based on the insights gathered from existing literature, this study proposes the utilization of STFT (Short Term Fourier Transform), LASSO (Least Absolute Shrinkage and Selection Operator), and EHO (Elephant Herding Optimization) as feature extraction and dimensionality reduction techniques. The research also compares the traditional machine learning classifiers such as DFA, NBC, GMM, and SVM (RBF) with integrated metaheuristic classifiers including PSO-GMM, Firefly-GMM, and Flower Pollination Optimization with GMM. The materials and methodology are discussed in the upcoming section.

## Materials and methodology

### Dataset

In this research, we have used a publicly accessible dataset to classify cases of colon cancer, provided by Alon et al.^33^. The dataset contains 2000 genes. There are 62 samples total; Class 1 represents the tumour class with 40 samples, and Class 2 represents the healthy class with 22 samples. Table 2 summarises the dataset's details in tabular form.

**Table 2:** Dataset details.

Dataset	Number of genes	Class 1 (cancer)	Class 2 (healthy)	Total samples
Colon cancer^33^	2000	40	22	62

### Workflow of the research

The microarray gene expression data generation involves numerous steps, starting with obtaining an RNA sample from the suspected colon cancer area cells. As explained by Sakyi et al.^34^, isolating RNA uses techniques like phenol–chloroform extraction. After obtaining the RNA sample, a process called reverse transcription is performed to convert RNA into complementary DNA (cDNA). The cDNA will be labelled with fluorescent dyes and other markers. The labelled cDNA is hybridized onto microarray slides containing thousands of known DNA sequences that correspond to genes of suspected colon cancer area. After hybridization, the microarrays are scanned to detect the fluorescence signals, which indicate the abundance of each gene's mRNA in the original sample. At last, the raw fluorescence intensity data are processed and analyzed to generate a microarray gene expression matrix. The rows of the microarray gene expression matrix represent a gene and each column represents a sample.

**Figure 1:**
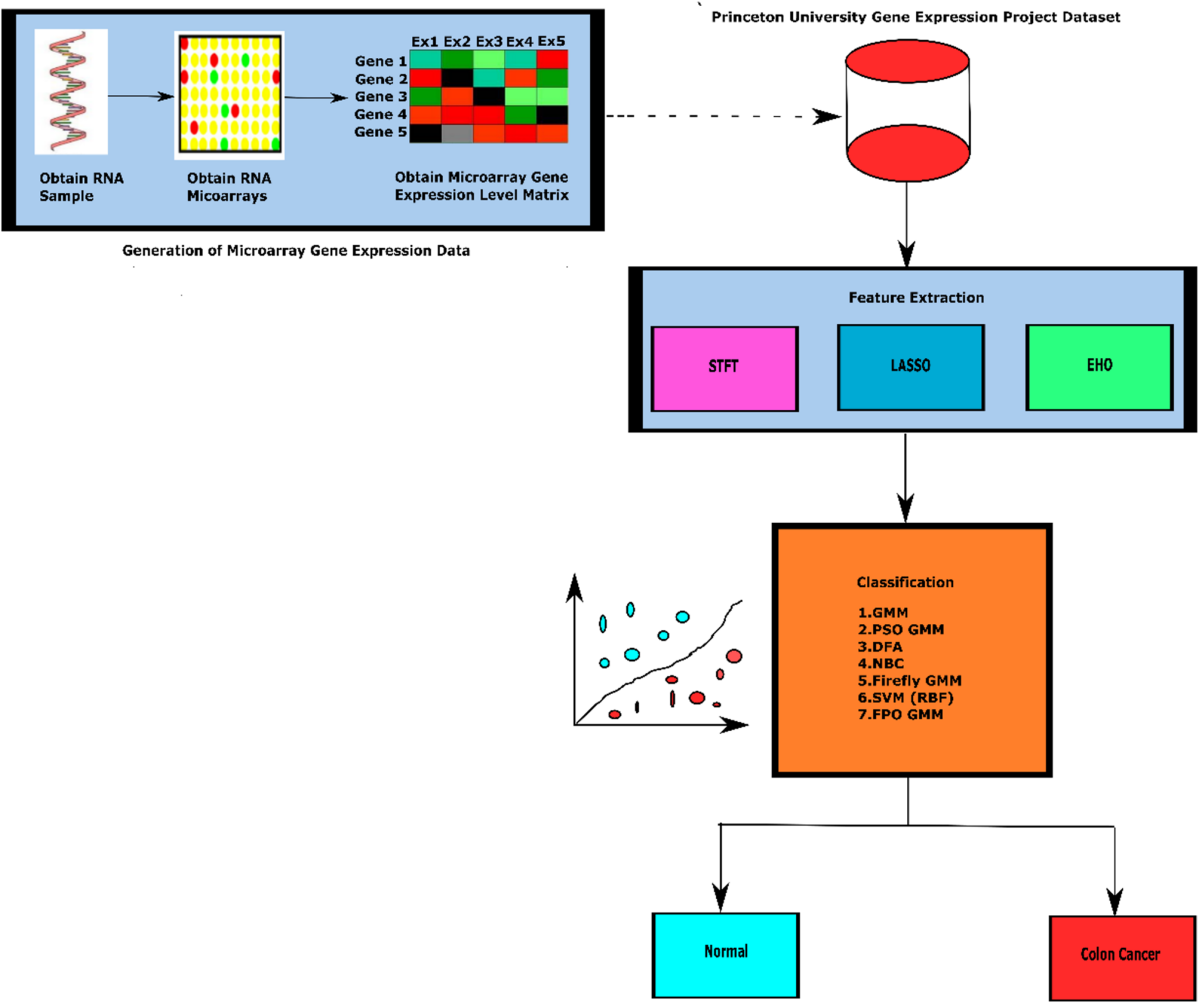
The schematic workflow of the research starts with the Colon cancer microarray gene expression dataset extracted from RNA microarrays. Then, feature extraction is performed on the microarray gene expression dataset using STFT, LASSO and EHO techniques. Further, Colon cancer classification is performed using machine learning and metaheuristic-integrated machine learning classifiers.

The microarray gene expression matrix provides valuable insights into the expression levels of thousands of genes simultaneously. It helps to identify significant patterns and differences in gene expression across diverse conditions and experimental groups. The major steps involved in the generation of microarray gene expression data along with the overall workflow of the research work are illustrated in Fig. 1. In the next section, the details about various feature extraction methods adopted in the research are discussed.

### Feature extraction methods

The feature extraction in this research is performed by utilizing the methods detailed in STFT (Gupta et al.^35^), LASSO (Tibshirani et al.^36^) and EHO feature extraction techniques (Wang et al.^37^).The STFT is nothing but a windowed Fourier transform that is used to capture time-varying frequency components in gene expression data. It enables the analysis of dynamic gene expression patterns over time intervals. LASSO employs regularization to simultaneously perform feature selection and data shrinkage. This technique effectively identifies the subset of genes with significant expression levels and mitigates the overfitting problems during microarray data analysis. On the other hand, EHO is a metaheuristic approach that is inspired by the herding behaviour of elephants. This nature-inspired extracts and also optimizes the features in the gene expression data, providing unique insights into gene interactions.

#### Feature extraction using STFT

A frequency domain study of the data over a brief time span is called the Short-Time Fourier Transform (STFT). By extracting pertinent and helpful features, the STFT can reduce the data dimension and extract relevant features when applied to microarray gene data. Gupta et al.^38^ have used STFT to do QRS Complex Detection. According to the authors, STFT is helpful in giving researchers a time–frequency representation of the data, enabling them to examine how variations in gene expression levels occur over various frequency components and over time. With STFT, a localised representation of frequency content over time can be obtained by identifying particular time intervals where particular frequency components are prominent. This STFT feature is helpful in finding genes that show temporal patterns active during particular time intervals for microarray gene expression research. Additionally, by identifying the crucial genes or gene clusters connected to particular frequency components, STFT lowers dimensionality. The Blackman window^39^ that reduces spectral leakage serves as the windowing function for STFT calculations. Therefore, by identifying the frequency patterns and underlying correlations in the dataset, STFT can offer biological insights. The computation of STFT is expressed as

1 $$X\left(m,w\right)= {\sum_{n=-\infty }^{\infty }}x\left[n\right]w[n-m]{{e}^{-jwn}}$$

Here, *x* [*n*] represents the input data having length *N* with *n* = 0, 1, 2, … *N* − 1. The STFT window is represented by *w*[*n*] having length *M* with *m* = 0, 1, 2, … M − 1, j = √ (− 1) denotes the complex number. The Blackman window is given by the expression,

2 $$w\left[n\right]=0.42-0.5\text{cos}\left(\frac{2\pi n}{M}\right)+0.08\text{cos}\left(\frac{4\pi n}{M}\right), 0\le n\le N-1$$

In essence, STFT would be able to extract features from the microarray gene expression data and capture significant time-varying patterns that allow the detection of dynamic gene expression changes crucial for understanding inherent biological processes. However, STFT often exhibits its inability to effectively handle high-dimensional data and multicollinearity. This can lead to inadequate performance or overfitting issues. Here we discuss the significance of LASSO which can overcome these issues by imposing a penalty on the absolute size of regression coefficients.

#### Feature extraction using LASSO regression

LASSO is an extension of ordinary least squares (OLS) regression introduced by Tibshirani et al.^40^. It works by minimizing the sum of squared residuals while imposing a constraint on the sum of the total values of the regression constants. The estimated regression coefficients in LASSO can be characterized as follows:

3 $$\beta =argmin\left\{{\sum }_{i=1}^{n}{({x}_{i}-{\sum }_{j=1}^{p}{\beta }_{j}{y}_{ij})}^{2}\right\}+\lambda {\sum }_{j=1}^{p}\left|{\beta }_{j}\right|$$

Here ‘n’ represents the number of observations in the dataset and ‘p’ represents the number of predictor variables or features in the dataset. The variable ${x}_{i}$ is the observed value in the ith observation, ${y}_{ij}$ is the predicted value in the ith observation and jth prediction, ${\beta }_{j}$ represents the regression coefficient associated with the jth predictor. The regularisation values are represented by, a dimensional vector that is also designated as the penalty term. The $\lambda$ variable contains the LASSO estimation values for the slope coefficients. It is reasonable to assume that the response variable has a mean of 0 without sacrificing generalizability. Also, the covariates act as predictors having a variance of 1 and a standardised mean of 0. When the L1 regularization is applied to the constants, a feature emerges: the coefficients tend to approach zero as λ grows, and for sufficiently large λ, some are precisely reduced to zero. Because of this special quality, the LASSO can choose models and produce predictable results. Thus, LASSO has effectively performed the feature selection by reducing the risk of overfitting in microarray gene expression data analysis. However, LASSO tends to select only a subset of features, potentially discarding relevant predictors that are correlated with the selected ones. Here, an Elephant Herding Optimization (EHO) can address this limitation by offering a nature-inspired optimization approach that explores a wider range of feature combinations, potentially capturing synergistic interactions among genes and providing a more comprehensive understanding of the underlying biological mechanisms in microarray gene expression data.

#### Feature extraction using elephant heard optimization

EHO is grounded on the food searching behaviour of elephants, is introduced in Wang et al.^41^. In the EHO metaheuristic algorithm, the location of each elephant *i* is iteratively updated as follows.

4 $${p}_{i}^{new}= {p}_{i}^{old}+ \Upsilon \left({p}_{best}- {p}_{i}^{old}\right)\times {rand}_{1}$$

Here ${p}_{best}$ is the global best and $\Upsilon$ indicates the control variable, $\Upsilon$ ∈ [0, 1], *rand*_*1*_ refers to the random number, ${rand}_{1}$ ∈ [0, 1]. The global best, ${p}_{best}$ is updated iteratively in the following manner.

5 $${p}_{best}^{new}=\updelta \times {p}_{center}$$

6 $${p}_{center}= \frac{1}{n}\times \sum_{i=1}^{n}{p}_{i}$$

where δ ∈ [0, 1] is another control parameter. In addition, the worst position is changed according to the following equation.

7 $${p}_{worst}= {p}_{min}+ \left({p}_{max}- {p}_{min}+1\right)\times rand$$

Here ${p}_{min}$ and ${p}_{max}$ mentions the minimum and maximum position values available in the solution space. In this way using the EHO's exploration of the solution space beyond sparse representations helps in mitigating the risk of overlooking to the important features, but at the same time optimizing the model performance to avoid overfitting problems during classification. In the next section, a statistical analysis of the extracted features obtained from STFT, LASSO, and EHO is analysed in detail.

### Statistical analysis of the extracted features using STFT, LASSO, and EHO

To ensure that the feature extracted data reduces the dimensionality and retains the significant characteristics of the original microarray data, we delve into a comprehensive statistical analysis. For both the normal and cancerous classes, a comparison of the extracted features is performed through various statistical parameters: Mean, Variance, Skewness, Kurtosis, Pearson Correlation Coefficient (PCC), and Canonical Correlation Analysis (CCA). The analysis presented in Table 3, gives a clear picture of whether the adopted feature extraction methods can preserve the crucial properties of the microarray genes data within each class. The analysis serves as the validation for the subsequent classification performance and interpretations.

**Table 3:** Statistical analysis of colon cancer micro array gene after bio inspired features.

Statistical parameter	STFT		LASSO regression		EHO	
	Normal	Cancer	Normal	Cancer	Normal	Cancer
Mean	6.43E + 03	7.29E + 03	0.0060	0.0545	0.0967	0.8288
Variance	5.02E + 07	6.41E + 07	1.72E−07	1.23E−07	9.8956E−05	0.0085
Skewness	1.85E + 00	1.74E + 00	− 2.40E−05	0.0103	− 3.0884	− 0.5309
Kurtosis	1.14E + 01	1.11E + 01	− 1.1999	−1.1904	9.9041	11.8288
PCC	3.33E−02	1.55E−02	− 0.14286	− 0.07692	0.0052	− 0.0073
CCA	0.5356		0.5843		0.4230	

*The mean value indicates the centre point of the gene expression data cluster along with variance indicating the dispersion of data from the centre point. Skewness and Kurtosis values compare the gene data distribution with the Gaussian data distribution. PCC reveals the internal association of gene expression levels inherent in the data. CCA examines the correlation of the data across cancer classes.

The statistical parameter ‘Mean’ is the average value of the set of data values in the dataset. It represents the average expression levels of genes across different samples. Due to the detailed frequency information and mathematical characteristics (without scaling and normalization), STFT provides very high mean values of 6.43E + 03 and 7.29E + 03 compared to LASSO and EHO. The mean values of LASSO and EHO are low because these are normalized methods with sparsity and compression. Variance measures the spread or dispersion of the values in the dataset. In gene expression, variance indicates how much individual expression levels deviate from the mean expression level. Similar to the mean values, the variance of the STFT data is very high when compared to LASSO and STFT methods. High variance suggests that the expression levels vary widely across samples, while low variance indicates that the expression levels are similar across samples.

Skewness measures the asymmetry of the distribution of values compared to normal distribution in the dataset. A positive skewness indicates that the distribution is skewed to the right (i.e., the tail of the distribution extends more to the right), while a negative skewness indicates that the distribution is skewed to the left. From Table 3, the STFT is a right-skewed distribution, meaning there are more samples with higher expression levels for this particular feature compared to lower expression levels. LASSO exhibits nearly a symmetrical distribution with skewness values close to zero. EHO exhibits strong negative skewness in the case of Normal data and a light negative skewness for Cancer data, suggesting a left-skewed distribution of expression levels. Kurtosis measures the peakedness or flatness of the distribution of values in a dataset compared to a normal distribution. High kurtosis indicates a sharp peak (leptokurtic), while low kurtosis indicates a flat peak (platykurtic). The extracted features using STFT and EHO are leptokurtic, meaning the distribution has heavier tails and more extreme values than a normal distribution. LASSO is platykurtic, revealing that the distribution has lighter tails and fewer extreme values than a normal distribution.

**Figure 2:**
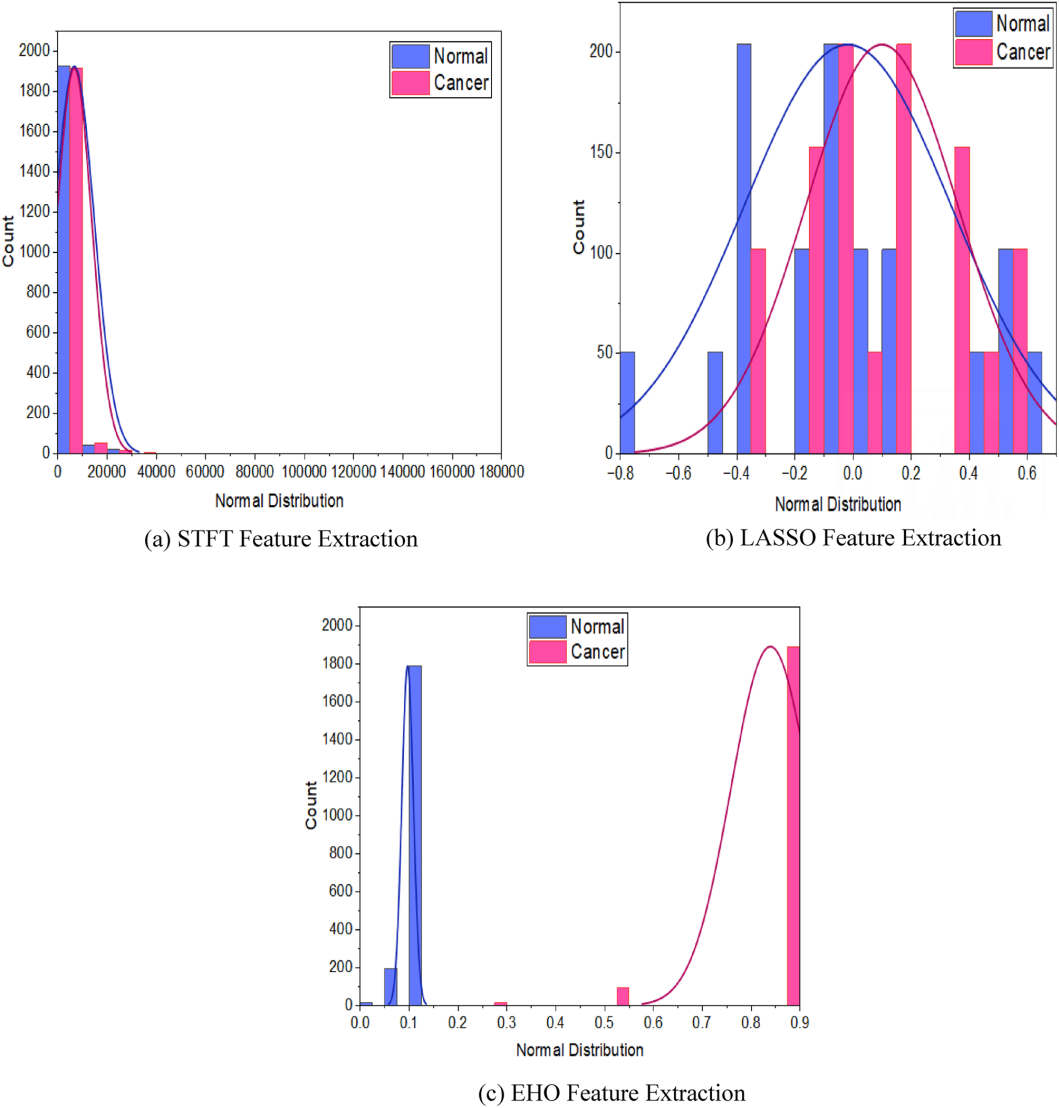
Data distribution plots for various feature extraction methods. The data distribution after STFT feature extraction is positively skewed reporting more correlation among the adeno and meso classes. The data distribution after LASSO feature extraction illustrates two differently behaved Non-Gaussian distributions with different statistics parameter values and also shows a moderate correlation among the classes. The data distribution for EHO feature extraction depicts minimum correlation among the classes, with distinct Non-Gaussian distributions segregated to their extremities.

PCC measures the linear relationship between two variables. In gene expression analysis, PCC is used to quantify the degree of linear association between the expression levels of two genes across different samples. A value close to 1 indicates a strong positive correlation, a value close to − 1 indicates a strong negative correlation and a value close to 0 indicates no linear correlation. For both the normal and cancer classes, STFT feature extraction shows a weak positive linear relationship, LASSO exhibits a weak negative linear relationship, and EHO shows no linear relationship. The CCA is a multivariate statistical technique used to analyze the relationship between two sets of variables. In gene expression analysis, CCA is used to identify linear relationships between two cancer classes. CCA finds linear combinations of the features that maximize the correlation between the two sets of cancer classes. A positive correlation among the classes is observed in STFT, and LASSO methods, whereas a weak positive correlation is reported in the EHO feature extraction method. The CCA is thus a crucial statistical parameter for understanding the associations between different cancer class feature that can help to identify patterns and underlying biological processes in the data. All these observations can bevisualized in the Fig. 2 which shows the data distribution of STFT, LASSO and EHO feature extraction methods.

**Figure 3:**
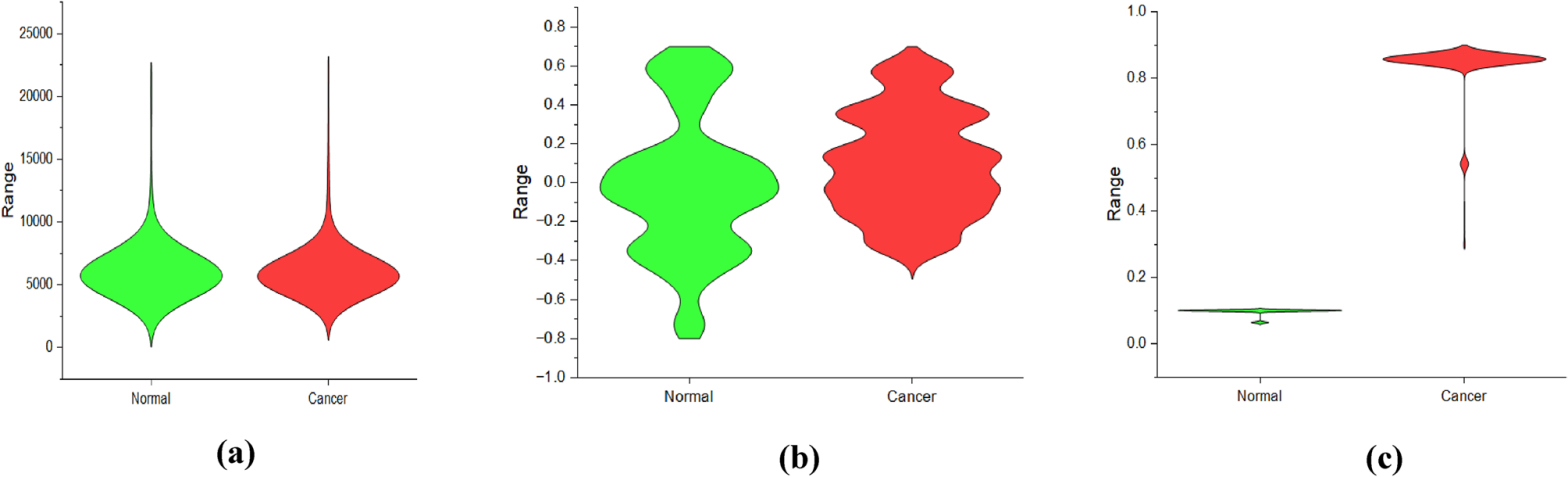
Violin plots for various feature extraction methods. (**a**) STFT feature extraction, (**b**) LASSO feature extraction, (**c**) EHO feature extraction LASSO and EHO appear to capture more variability and distinct patterns compared to STFT.EHO shows particularly promising results in separating normal and cancer classes based on the distribution of extracted features.

Up next, we compare the two class groups in our dataset using the Viloin plot shown in Fig. 3 to compare the distributions of numeric data across the two groups. A Violin plot that combines aspects of a box plot (median, quartiles, and outliers) with a kernel density plot (which shows the distribution of the data). The width of the violin at any given point represents the probability density of the data at that value. Therefore, wider sections indicate a higher probability density, while narrower sections indicate a lower probability density.

The violin plots are the same for STFT and this indicates that the distribution of features extracted using STFT is similar across both classes (normal and cancer). This suggests that STFT may sometimes fail to capture the differences in gene expression patterns between normal and cancer samples. The spread of the violin plot with more width for LASSO suggests that there is more variability in the distribution of features extracted using LASSO across both normal and cancer classes. This implies that LASSO is capable of capturing a wider range of gene expression patterns. Also, the normal class has more violin length compared to the cancer class reports that there may be more variability in the normal samples compared to the cancer samples for the features extracted using the LASSO method. In EHO, there is a spread in the width of the violin in certain regions suggesting variability in the distribution of features extracted using EHO across both normal and cancer classes. The non-overlapping of violin lengths portrays the significant differences in the distribution of features extracted using EHO between the two classes. This reveals that EHO is effective in capturing distinct gene expression patterns associated with normal and cancer samples. Thus, from all these statistical parameters it is clear that the extracted features are extremely relevant and significant for the upcoming cancer classification step.

## The classification approach

Once the features are extracted, the colon cancer classification is performed using machine learning and metaheuristic classifiers. In our research, we utilize a diverse set of seven classifiers to cast a wide net for the most suitable approach. The machine learning classifiers utilised are GMM, DFA, NBC, and SVM (RBF). These are probabilistic modelling used to identify clusters of similar gene expressions and patterns within the data. Further to uncover the potentially hidden dynamics inside the data, we employ the metaheuristic integrated machine learning classification namely PSO-GMM, Firefly-GMM, and FPO-GMM. By utilizing a diverse range of algorithms, we aim to capture the multifaceted nature of the data and identify the most accurate and robust approach for colon cancer classification.

### Gaussian mixture model (GMM)

The Gaussian Mixture Model is a popular unsupervised learning technique that groups together similar data points to perform tasks like image classification and pattern recognition. A set of Gaussian distributions are joined linearly in the PDF (Probability Density Function) of GMM, which makes the classification of the data easier. So the mixture classifier creates a probability distribution from microarray gene expression levels for both the classes with the help of a combination of Gaussian distributions. After probability distribution, the class prediction is based on the highest probability value (Bayes' theorem). For every class ‘c’, and feature ‘f’, GMM assumes that the data is drawn from a mixture of ‘G’ Gaussian coefficients. This can be expressed in the following way:

8 $$P\left(f\right|y=c)= \sum_{g-1}^{G}{M}_{cg}N (f |{ \Upsilon}_{cg} , { \text{C}\cdot }_{cg})$$

Here ${\text{M}}_{cg}$, ${\Upsilon}_{cg}, { \text{C}\cdot }_{cg}$ indicates coefficient of mixture, mean vector, and covariance matrix respectively, under mixture component ‘g’ in class ‘c’. The ${\text{M}}_{cg}$ signify the ratio of each constituent in the class. From training data, ${\text{M}}_{cg}$, ${\Upsilon}_{cg}, { \text{C}\cdot }_{cg}$ parameters are gained for each constituent in the class with ‘N’ as the total number of samples, total classes ‘C’, and i = 0,1,2,…N.

9 $${\Upsilon}_{c} = \frac{1}{{N}_{c}}\sum_{i=1}^{N}{{(y}_{i}=c)}_{1}. {f}_{i}$$

10 $$\text{C}\cdot =\frac{1}{N-G}\sum_{c=1}^{C}\sum_{i=1}^{N}{{(y}_{i}=c)}_{1} ({f}_{i} - {\Upsilon}_{c}){({f}_{i} - {\Upsilon}_{c})}^{T}$$

Also, $P (y=c)$ indicating prior probability is also obtained for every class ‘c’. $P (y=c)$ is given as:

11 $$p \left(y=c\right)= \frac{Number \; of \;samples \; with \;class \;c}{Total \;number \;of \;samples}$$

A new feature, ${f}_{new}$ classification is performed by likelihood estimation under mixture model using Bayes' theorem in the following way.

12 $$p\left(y=c|{f}_{new}\right)\propto p \left(y=c\right)\cdot p\left({f}_{new}|y=c\right)$$

13 $$p\left({f}_{new}|y=c\right) = \frac{1}{{\left(2\pi \right)}^{D/2}{\left|\text{C}\cdot \right|}^{1/2}}\text{ exp }\left(-\frac{1}{2}{\left({f}_{new}- {\Upsilon}_{k}\right)}^{T}{\text{C}\cdot }^{-1}({f}_{new}- {\Upsilon}_{k})\right)$$

Here ‘D’ represents the dimensionality of the data, that is nothing but the number of features.

### Particle swarm optimization-GMM (PSO-GMM)

PSO utilizes the collective intelligence of a "swarm" to optimize the classification performance. Consider a flock of birds searching for food, constantly refining their flight paths based on individual discoveries and interactions. PSO can effectively perform classification by searching for the most clustered and hidden subset of genes from the large pool of gene expression data. Moreover, PSO can handle the complex and non-linear relationships among feature-extracted gene expressions. A combination of PSO with GMM (PSO-GMM) can intricate relationships that might be missed by static GMM. The technique potentially provides a more accurate cluster identification and classification. Here's the crux of PSO-GMM classification, Nair et al.^42^.

14 $${v}_{i}(t+1) = w{v}_{i}(t) + {c}_{1}{r}_{1}({pbest}_{i} - {x}_{i}(t)) + {c}_{2}{r}_{2}(gbest - {x}_{i}(t))$$

where ${v}_{i}(t)$ is the velocity of particle i at iteration t, w is the inertia weight, c_1_ and c_2_ are constriction coefficients, r_1_ and r_2_ are random numbers between 0 and 1, ${pbest}_{i}$ is the best position found by particle i so far, $gbest$ is the best position found by any particle in the swarm, ${x}_{i}(t)$ is the current position of particle i.

After application of PSO, the search space will be optimized and data distribution will be changed. Now, PSO-GMM follows the expressions from Eqs. (8–13) to perform the final classification. PSO-GMM is a methodology that can unlock hidden patterns in gene expression data and improves classification accuracy by avoiding overfitting issues.

### Firefly GMM

The Firefly Algorithm (FA) is a nature-inspired metaheuristic that mimics the flashing behaviour of fireflies to solve optimization problems. Consider a firefly flitting through the night, its brightness representing its "fitness" in finding mates or food. So, brighter fireflies attract others and guide them towards better locations. Using this behaviour, FA can segregate most of the relevant features and escape from the local optima of the microarray data. This exploration and search mechanism of the solution space results in reliable and robust classification outcomes. Here's the simplified representation of the attraction between fireflies:

15 $${I}_{i} ={{I}_{i}}^{0}{e}^{(-\gamma {r}_{ij}^2)}$$

where ${I}_{i}$ is the attractiveness, ${{I}_{i}}^{0}$ is the initial attractiveness, γ is the light absorption coefficient, ${r}_{ij}$ is the distance between fireflies i and firefly j. Fireflies move towards brighter neighbours, gradually refining their positions towards better solutions. The movement of a firefly i towards a brighter firefly j is determined by the attractiveness and randomness given by

16 $${{x}_{i}}^{t+1}= {{x}_{i}}^{t}+ \beta \left({{I}_{j}}^{t}- {{I}_{i}}^{t}\right)+ \alpha (rand\left( \right)-0.5)$$

Here, ${{x}_{i}}^{t+1}$ is the new position of firefly i at time step t + 1, ${{x}_{i}}^{t}$ is the current position of firefly i at time step t, β is the attraction coefficient, α is the randomization parameter, and $rand\left(\right)$ ∈ [0, 1], is a generated random number.

The collaboration of Firefly with GMM can potentially uncover intricate relationships in gene expression data that might be missed by standard GMM. Firefly combined with GMM can also overcome the limitations of the dimensionality problem. The Firefly GMM thus gives a clustered and segregated data distribution to GMM for classification by varying the mean, variance and other statistical parameters of the extracted features.

### Detrended fluctuation analysis (DFA)

Detrended fluctuation analysis (DFA) efficiently solves problems involving complex and non-stationary data. DFA captures both short and long-range correlations based on how the data fluctuates around the data distribution with the help of a scaling exponent to classify the data. The scaling nature of DFA algorithm is described by the root mean square fluctuation of the integrated time series and detrended input data. For DFA, the inputs are analysed in the following way.

17 $$y\left(k\right)={\sum }_{i=1}^{k}[B\left(i\right)-\overline{B }]$$

where $B\left(i\right)$ and $\overline{B }$ are the ith sample of the input data and mean value of the input data respectively, thus $y\left(k\right)$ denotes the estimated value of the integrated time series. Now, the fluctuation of integrated time-series and detrended data for a window with the scale of ‘n’ is determined by

18 $$F\left(n\right)=\sqrt{\frac{1}{N}{\sum }_{k=1}^{N}[y\left(k\right)-{y}_{n}(k){]}^{2}}$$

Here, ${y}_{n}(k)$ is the kth point on the trend computed using the predetermined window scale, and ‘N’ is the normalization factor.

Therefore using Detrended Fluctuation Analysis (DFA) as a classifier for microarray gene expression data can effectively capture the long-range correlations present in microarray gene expression data. Unlike traditional methods focusing on short-range correlations, DFA evaluates the scaling behaviour of fluctuations across different time scales. This capability is particularly valuable in gene expression data analysis, where genes may exhibit complex patterns of co-regulation and interactions across various biological processes and time scales.

### Naive Bayes classifier (NBC)

NBC is a probabilistic classification algorithm based on the Bayes theorem and feature independence assumption. This straightforward assumption allows Naive Bayes classifiers to efficiently handle large feature spaces, making them computationally efficient and scalable for microarray data analysis. NBC starts by calculating the posterior probability of a class using the prior probability and likelihood. For a given class C and extracted features x_1_, x_2_… x_n_, posterior probability $p\left(C|{x}_{1},{x}_{2},\dots ., {x}_{n}\right)$ is expressed in Fan et al.^43^ as:

19 $$p\left(C|{x}_{1},{x}_{2},\dots ., {x}_{n}\right)= \frac{p\left(C\right).p({x}_{1},{x}_{2},\dots ., {x}_{n}|C)}{p({x}_{1},{x}_{2},\dots ., {x}_{n})}$$

For class C, $p\left(C\right)$ represents the prior probability, $p({x}_{1},{x}_{2},\dots ., {x}_{n}|C)$ is the likelihood, and $p({x}_{1},{x}_{2},\dots ., {x}_{n})$ is the evidence probability. As mentioned, in the Naive Bayes approach, the features are conditionally independent for the class. This assumption simplifies the calculation of the likelihood as follows:

20 $$p({x}_{1},{x}_{2},\dots ., {x}_{n}|C) = p({x}_{1}|C) . p({x}_{2}|C) \dots p({x}_{n}|C)$$

where $p({x}_{i}|C)$ is the probability of feature ${x}_{i}$ of the class C. The $({x}_{i}|C)$ is estimated from the fraction of class C training examples with the feature value ${x}_{i}$. Then the prior probability (*C*) can be estimated as the fraction of training examples belonging to class C. Finally, to predict the class label for the features ${x}_{i}$, the algorithm calculates the posterior probability for each class and assigns the instance to the class with the highest probability. Thus using a Naive Bayes classifier for microarray gene expression data can efficiently handle large amount of data because they assume independence between features given the class label, which greatly reduces computational complexity.

### Support vector machine (radial basis function)

Support Vector Machine with a Radial Basis Function (SVM RBF) can handle complex, non-linear relationships between gene expression levels. SVM can effectively handle non-linear separability by mapping the input data into a high-dimensional feature space, where non-linear relationships become linearly separable. This is possible by constructing decision boundaries between normal and cancerous samples by handling non-linear relationships effectively. RBF is the kernel used in SVM to perform the nonlinear mapping of the input features into a higher-dimensional. The RBF kernel ${K}_{RBF}\left(x,z\right)$ that is used to compute the similarity between feature vectors in the input space is given by:

21 $${K}_{RBF}\left(x,z\right)=\text{exp}\left(-\frac{{\Vert x-z\Vert }^{2}}{2{\sigma }^{2}}\right)$$

where σ is the kernel width parameter that regulates the influence of each training sample, and |x − z| is the Euclidean distance between feature extracted vectors ‘x’ and ‘z’. The SVM RBF classification decision function is also described as a linear combination of the kernel evaluations between the input feature vector and the support vectors with the bias term, as performed by Zhang et al. ^48^. The decision function ${f}_{RBF}\left(x\right)$ is given by:

22 $${f}_{RBF}\left(x\right)=\sum_{i=1}^{N}({\alpha }_{i}{y}_{i}){K}_{RBF}({x}_{i, }x)+b$$

Here ${y}_{i},$ and ${\alpha }_{i}$ are Lagrange multipliers and class labels respectively associated with each support vector. The ${K}_{RBF}\left(x,z\right)$ computes the computes the similarity or distance between two feature-extracted vectors ${x}_{i}$ and $x$. $b$ is the bias term that shifts the decision boundary away from the origin allowing the SVM to classify data points that may not be separable by a hyperplane in the original feature space.

### Flower pollination optimization (FPO) with GMM

FPO is a metaheuristic optimization algorithm inspired by the pollination behaviour of flowering plants. In nature, pollination happens through two major forms: abiotic and biotic. The biotic cross-pollination is observed in 90% of the cases where the insects are supporting the pollination process. The insects thus take long distance steps, in which the motion can be drawn from a levy distribution. The FPO starts with the discovery of solution space and subsequent movements.The insect motion and the levy distribution are expressed in the following steps as provided in Yang et al. ^49^.

23 $${{x}_{i}}^{t+1}= {{x}_{i}}^{t}+ \delta L \left(\lambda \right) ({g}_{best}- {{x}_{i}}^{t})$$

where ${{x}_{i}}^{t}$ is a pollen representing the solution vector ${x}_{i}$ at the $t$th iteration, and ${g}_{best}$ is the global best solution discovered so far. The step size is denoted by factor, and $L \left(\lambda \right)$ is the Levy flight step size denoting the success of pollination.

The Levy distribution is given by:

24 $$L \sim \frac{\lambda \Gamma\left(\lambda \right)\text{sin}\left(\pi \lambda /2\right)}{\pi } \frac{1}{{S}^{1+\lambda }}, ({s} \gg {s}_{0} >0)$$

Where $\Gamma\left(\lambda \right)$ the step size for is large steps where $({s}>0)$, drawn from a gamma distribution. However, for smaller pseudo-random steps, that correctly follow Levy distribution, the $s$ value is drawn from two Gaussian distributions U and V (Mantegna algorithm ^50^),

25 $$s= \frac{U}{{\left|V\right|}^{\left(1/\lambda \right)}}, U \sim N \sim N \left(0,{\sigma }^{2}\right), V \sim N (\text{0,1})$$

Here, $U$ is drawn from a normal distribution with mean = 0 and variance = ${\sigma }^{2}$, and $V$ is drawn from a normal distribution with mean = 0 and variance = 1. The variance is given by

26 $${\sigma }^{2}= {\left\{ \begin{array}{ccc}\frac{\Gamma (1+\lambda )}{\lambda \Gamma [(1+\lambda )/2] }& .& \frac{\text{sin}\left(\pi \lambda /2\right)}{{2}^{(\lambda -1)/2}}\end{array}\right\}}^{1/\lambda }$$

The remaining 10% of the abiotic pollination observed in the plant and flower community is regarded as a Random Walk, that sometimes brings out solutions from the unexplored search space ${x}_{i}$.

27 $${{x}_{i}}^{t+1}= {{x}_{i}}^{t}+ \varepsilon ({{x}_{j}}^{t}- {{x}_{k}}^{t})$$

Here, $$ {{x}_{i}}^{t}$$, and $${{x}_{k}}^{t}$$ are considered to be pollen transformations within the same plant species, from the same population. $$\varepsilon$$ is the step for the random walk drawn from the uniform distribution [0,1].

Thus, FPO allows for a global search of the solution space, which is crucial for effectively exploring the high-dimensional feature space of gene expression data. FPA identifies the most relevant genes that can contribute to the classification task and reduces the dimensionality of the feature extracted data. FPA also changes the means, covariance, and mixing coefficients of data distribution, so that GMM can perform better with the optimized data that contains complex and non-linear relationships among genes. The next section discusses the selection of classifier targets for the Adeno and Meso classes.

### Selection of target

Selecting the target is a crucial step in defining the objective of the classification methodology. The clear definition of target improves the classifier model's predictive power by focusing on the most informative aspects of the data. The noise, outliers, and nonlinearity aspects of the data decide the selection of classifier targets. Moreover, the dataset used in this research is imbalanced and selecting the target must be strategized to deliver the maximum classifier performance. The binary classification performed in this research where the classification of feature extracted microarray data samples are classified into normal and colon cancer classes. Therefore two targets are selected namely ${\text{T}}_{{\varvec{N}}}$ and ${\text{T}}_{{\varvec{C}}}$. For a normal class feature set of N elements, the class target for the Normal class is defined as:

28 $$\frac{1}{N} \sum_{i=1}^{N}{M}_{i} \le {T}_{N}$$

where ${M}_{i}$ is the average of the feature extracted vectors of the Normal class, and ${T}_{{\varvec{N}}}$ target follows the constraint ${T}_{{\varvec{N}}}$∈ [0, 1]. For a Colon Cancer class feature set of M elements, the class target for the Colon Cancer class is defined as:

29 $$\frac{1}{M} \sum_{j=1}^{N}{M}_{j} \le {T}_{C}$$

where ${M}_{i}$ is the average of the feature extracted vectors of Colon cancer class, and ${T}_{C}$ target follows the constraint ${T}_{C}$ ∈ [0, 1]. The Eucledian distance between the Targets of the binary class must also follow the constraint:

30 $$\Vert {\text{T}}_{N} - {\text{T}}_{C} \Vert \ge 0.5 $$

Based on the Eqs. (28–30), the class targets ${T}_{N}$ and ${T}_{C}$ are chosen as 0.85 and 0.1, respectively. The classifier performance will be monitored with the help of MSE criteria. The next section discusses the training and testing of classifiers.

### Training and testing of classifiers

Before moving to the final classification step, training of classifiers is performed utilizing the labelled microarray dataset to adjust the classifier model's parameters. This step enables the learning of complex patterns and relationships in the gene expression data and optimizes the classifier's performance by minimizing the discrepancy between predicted and actual outcomes. After training, testing evaluates the trained classifier's performance on an independent dataset not used during training. So the testing phase assesses the model's generalization ability and provides insights into its effectiveness on other datasets. Mean Square Error (MSE) serves as a critical evaluation metric during both the training and testing phases. In training, MSE quantifies the disparity between predicted and actual gene expression values, guiding the optimization process to minimize prediction errors. During testing, MSE provides insights into the model's predictive accuracy and its ability to generalize to unseen data. Minimizing MSE ensures that the classifier effectively captures the underlying relationships within the gene expression data, enhancing its predictive performance. The MSE is given by calculating the average squared difference between the predicted values and the actual values in a dataset.

31 $$MSE= \frac{1}{N}{\sum }_{j=1}^{N}({A}_{j}-{P}_{j}{)}^{2}$$

Here ‘N’ is the total number of extracted features, ${A}_{j}$ represents the actual gene expression value of jth instance, and ${P}_{j}$ represents the predicted gene expression value of the jth instance. A lower MSE indicates better performance, as it signifies smaller discrepancies between predicted and actual values. Conversely, a higher MSE suggests poorer performance and potentially larger prediction errors. Thus, MSE acts as a parameter that continuously checks the performance of the classifier.

We also perform K-Fold Cross Validation as performed in Fushiki et al.^44^ to validate the classifier model. In this approach, the dataset is divided into K subsets (folds), with each fold serving as a validation set while the remaining data is used for training. This process is repeated K times, ensuring that each data point is used for validation exactly once. By averaging the performance metrics across multiple validation iterations, K-Fold Cross Validation provides a more reliable estimate of the classifier's performance and helps mitigate overfitting, thus enhancing the generalization ability of the model. In this research, we have varied K value in the range 5–20, and is finally the value is fixed with 10, as higher values are providing similar results.

In this research for the binary classification problem of colon cancer data, the confusion matrix is described as shown in Table 4. It is used to describe the performance of a classification model during the training and testing phase. The confusion matrix has four possible combinations of predicted and actual class labels: True Positives (TP), True Negatives (TN), False Positives (FP), and False negatives (FN). These values help to evaluate the overall classifier performance metrics like Accuracy, F1 Score, Error Rate, MCC, and Kappa.

**Table 4:** Confusion matrix.

	Predicted normal	Predicted cancer
Actual normal	TN	FP
Actual cancer	FN	TP

TP: Samples that are correctly classified as colon cancer.

TN: Samples that are correctly classified as normal.

FP: Samples that are incorrectly classified as colon cancer when they are actually normal.

FN: Samples that are incorrectly classified as normal when they are actually colon cancer.

Table 5 shows the obtained training and testing MSE of classifier for various feature extraction methods. The training MSE is reported between 10^−01^ and 10^−9^_._ The testing MSE is reported between 10^−5^ to 10^−8^.The training process is evaluated with 2000 iterations. The FPO-GMM with STFT feature extraction attained the lowest training and testing MSE of 7.29 × 10^–09^ and 6.44 × 10^–07^, respectively. For LASSO feature extraction, SVM (RBF) attained the lowest training and testing MSE of 1.6 × 10^–07^ and 9 × 10^–08^, respectively. For EHO feature extraction SVM (RBF) classifier attained the lowest training MSE of 1.67 × 10^–07^ and the FPO-GMM classifier attained the lowest testing MSE of 9 × 10^–08^.

**Table 5:** Training and testing MSE of classifiers for STFT, LASSO and EHO feature extraction techniques.

Classifiers	STFT feature extraction		LASSO feature extraction		EHO feature extraction	
	Training MSE	Testing MSE	Training MSE	Testing MSE	Training MSE	Testing MSE
GMM	1.02 × 10^–05^	1.44 × 10^–05^	1.02 × 10^–05^	7.84 × 10^–06^	2.25 × 10^–06^	2.25 × 10^–06^
PSO-GMM	1.02 × 10^–05^	9.01 × 10^–06^	4.84 × 10^–06^	6.25 × 10^–06^	1.44 × 10^–06^	1 × 10^–06^
DFA	4.36 × 10^–05^	2.35 × 10^–05^	1.3 × 10^–05^	1.44 × 10^–05^	2.89 × 10^–01^	2.24 × 10^–06^
NBC	4.36 × 10^–05^	6.08 × 10^–05^	6.25 × 10^–06^	3.24 × 10^–06^	2.25 × 10^–06^	2.25 × 10^–06^
Firefly-GMM	6.2 × 10^–05^	2.21 × 10^–05^	8.41 × 10^–06^	7.29 × 10^–06^	2.89 × 10^–06^	6.4 × 10^–07^
SVM (RBF)	7.84 × 10^–06^	1.44 × 10^–06^	**1.6 × 10**^**–07**^	**9 × 10**^**–08**^	**1.67 × 10**^**–07**^	2.5 × 10^–07^
FPO-GMM	**7.29 × 10**^**–09**^	**6.44 × 10**^**–07**^	1.44 × 10^–06^	3.6 × 10^–07^	2.25 × 10^–07^	**9 × 10**^**–08**^

Significant values are in bold.

Based on the observations from Table 5, the classifier parameters are selected for the various employed classifiers in this research as provided in Table 6.

**Table 6:** Parameter selection of the employed classifiers.

Classifiers	Parameters
GMM	$\text{Coefficient of mixture}-{M}_{cg}$, Mean vector-${\Upsilon}_{cg}, { \text{C}\cdot }_{cg}$-Covariance matrix are initialized to zero. Test point likelihood probability = 0.1, Cluster probability = 0.5, Convergence rate = 0.6, Convergence Criteria = MSE of 10^–7^
PSO-GMM	Population Size, N = 200, Inertia Weight ($w$) = 0.7, Constriction coefficients: c1 = 1.5 and c2 = 1.5, Random numbers: r1 and r2 ∈ [0, 1], Maximum Number of Iterations = 1000 or Convergence Criteria = MSE of 10^–7^
DFA	Window Scale (n) = 1.6, Polynomial Order = 1, Normalization Factor (N) = 1.6, Degree of window overlap = 50%, Convergence Criteria = MSE of 10^–7^
NBC	Smoothing factor, α = 0.06, Prior probabilities = 0.15, Distribution Assumption = Gaussian Naive Bayes, Convergence Criteria = MSE of 10^–7^
Firefly-GMM	Population Size, N = 200, Initial attractiveness ${I}_{0}$ = 1, Randomization Parameter (α) = 0.1, Attraction coefficient β = 0.6, Light absorption coefficient γ = 0.1, Distance between two fireflies r = Eucledian, Maximum Number of Iterations = 1000 or Convergence Criteria = MSE of 10^–7^
SVM (RBF)	Kernel width parameter (σ) = 0.1, Regularization Parameter (C) = 1, Class Weights (w) = 0.86, Bias, b = 0.01, Convergence Criteria = MSE of 10^–7^
FPO-GMM	Population Size, N = 200, Step Size ($\delta$) = 0.15, Pollination Rate (λ) = 1.5, Random walk step $\varepsilon$ ∈ [0, 1] (Uniform distribution), Switch Probability (ρ) = 0.65, Maximum Number of Iterations = 1000 or Convergence Criteria = MSE of 10^–7^

## Results and discussion

From the gene expression feature extracted data, 85% are used for training, and the remaining 15% are used for testing of models. The confusion matrix is a useful tool for assessing a machine learning model's performance in binary classification situations. The performance metrics for the binary classifier are based on the confusion matrix provided in Table 4. Through a comprehensive comparison across various feature extraction and metaheuristic and machine learning integrated classification algorithms, we strive to pinpoint the method that delivers the most accurate and consistent colon cancer classification based on gene expression data.

### Classifier performance

As previously indicated, the confusion matrix lists the model's predictions in comparison to the actual labels of the data. By examining the values in the confusion matrix, performance metrics including accuracy, precision, F1 score, MCC, error rate, and kappa are obtained. These metrics offer valuable insights into the sensitivity, specificity, and overall effectiveness of each classification technique. The classifier performance metrics and their expression from the confusion matrix are listed in Table 7.

**Table 7:** Classifier performance metrics.

Classifier performance	Expression from confusion matrix
Accuracy	$\text{Accuracy}=\frac{(\text{TN}+\text{TP})}{(\text{TN}+\text{FN}+\text{TP}+\text{FP})}$
F1 score	$\text{Precision}=\frac{\text{TP}}{\left(\text{TP}+\text{FP}\right)}$ $\text{Recall}=\frac{\text{TP}}{(\text{TP}+\text{FN})}$ $\text{F}1\text{ Score}=\frac{2\times \text{TP}}{(2\times \text{TP}+\text{FP}+\text{FN})}$
Error rate	$\text{Error rate}=\frac{(\text{FP}+\text{FN})}{(\text{TP}+\text{TN}+\text{FP}+\text{FN})}$
MCC	$MCC= \frac{(\text{TP}\times \text{ TN}-\text{FP }\times \text{ FN})}{\sqrt{TP+FP) \times (TP+FN) \times (TN+FP) \times (TN+FN)}}$
Kappa	$\text{Po}=\frac{(\text{TP}+\text{TN})}{(\text{TP}+\text{TN}+\text{FP}+\text{FN})}$ $\text{Pe}=\frac{(\text{TP}+\text{FP}) \times (\text{TP}+\text{FN})+(\text{FP}+\text{TN}) \times (\text{FN}+\text{TN})}{{(\text{TP}+\text{TN}+\text{FP}+\text{FN})}^{2}}$ $\text{Kappa}=\frac{(\text{Po}-\text{Pe})}{(1-\text{Pe})}$

*Po is the observed proportion of agreement, *Pe* is the proportion of agreement expected by chance.

A classifier's accuracy is determined by how well it recognises the class labels in a dataset. It is obtained by dividing the total number of examples in the dataset by the number of successfully identified instances. A classifier's accuracy can be further gauged by its F1 score, which combines recall and precision into a single statistic. The F1 score is computed as the harmonic mean of precision and recall. The value of the F1 score ranges from 0 to 1, with 1 denoting perfect precision and recall. Thus, F1 score is more considered over the accuracy, especially in the case of imbalanced datasets. The error rate of a classifier is the proportion of misclassified instances. MCC stands for “Matthews Correlation Coefficient”, which measures the quality of binary classification models. It considers true and false positives and false negatives which is particularly useful in situations where the classes are imbalanced.

The kappa statistic, also known as Cohen’s kappa, measures agreement between two raters or between a rater and a classifier. The agreement between the true and projected classes is measured using the kappa statistic, which takes into account the likelihood that the agreement is coincidental. The Kappa coefficient = 1 denotes perfect agreement, and any value below one can be interpreted as follows: There are five levels of agreement: poor (< 0.2), fair (0.2–0.4), moderate (0.4–0.6), good (0.6–0.8), and very good (0.8–1).The performance analysis of classifiers with STFT feature extraction is first discussed in Table 8.

**Table 8:** Performance analysis of classifiers in colon cancer detection from STFT feature extraction.

S.No.	Classifiers	Accuracy	F1Score	MCC	Error rate	Kappa
1	GMM	80.64516129	84.21052632	0.599404003	19.35483871	0.593886463
2	PSO-GMM	82.25806452	85.71428571	0.627341804	17.74193548	0.624035281
3	DFA	70.96774194	76.31578947	0.394460723	29.03225806	0.390829694
4	NBC	62.90322581	67.6056338	0.269679945	37.09677419	0.258064516
5	Firefly-GMM	67.74193548	74.35897436	0.310317338	32.25806452	0.309576837
6	SVM (RBF)	87.09677419	90	0.718181818	12.90322581	0.718181818
7	FPO-GMM	90.32258065	92.5	0.788636364	9.677419355	0.788636364

From the reported results in Table 8, it is evident that FPO-GMM has performed the best among all the other classifiers taken to consideration. An accuracy of 90.32% indicates that the classifier is performing well in correctly classifying instances. The high F1 Score of 92.5% suggests a good balance between precision and recall, which is crucial for imbalanced datasets. MCC of 0.7886 is also quite high, indicating a strong correlation between predicted and true classifications. The Kappa value of 0.7886 indicates that classifier's predictions are consistent with the true classifications, considering the potential for randomness in the classification process. PSO-GMM has reported better performed better when compared to Firefly-GMM due to their differences in their optimization capabilities and convergence behaviour. PSO is likely to explore the solution space more effectively, leading to higher performance metrics compared to the Firefly Algorithm. PSO's ability to efficiently search for optimal solutions contributes to better classification accuracy and an MCC of 0.6273 reflects a stronger agreement between predicted and true classifications. In contrast, Firefly Algorithm might struggle with convergence or effective exploration, resulting in lower performance metrics across the board.

SVM (RBF) achieved the second highest accuracy of 87.0967, F1 Score of 90, MCC of 0.7181, and Kappa of 0.7181 values. This is because SVM (RBF) is effective the handling the high-dimensional gene expression data and capturing the complex nonlinear relationships. GMM yielded moderate performance metrics as the assumption of Gaussian distributions led to a lower performance compared to SVMs. DFA showed lower performance compared to SVM and GMM, suggesting this classifier is not well-suited for capturing the intricate patterns in gene expression data. Naive Bayesian produced the lowest performance metrics due to the assumption of independence between features. This is because, Naive Bayes is simple and efficient and assumes independence between features, which is not true for gene expression data. The performance analysis of classifiers with LASSO feature extraction is next discussed in Table 9.

**Table 9:** Performance analysis of classifiers in colon cancer detection from LASSO regression feature extraction.

S.No.	Classifiers	Accuracy	F1 score	MCC	Error rate	Kappa
1	GMM	83.87097	87.17949	0.656355	16.12903	0.654788
2	PSO-GMM	85.48387	88.31169	0.696061	14.51613	0.692393
3	DFA	80.64516	84.21053	0.599404	19.35484	0.593886
4	NBC	87.09677	89.74359	0.725562	12.90323	0.723831
5	Firefly-GMM	83.87097	87.17949	0.656355	16.12903	0.654788
6	SVM (RBF)	96.77419	97.5	0.929545	3.225806	0.929545
7	FPO-GMM	93.5483871	95	0.859090909	6.451612903	0.859090909

From Table 9, it is evident that SVM (RBF) showed the best performance for LASSO based feature extraction method. A high accuracy of 96.77%, F1 Score of 97.5% indicate a strong performance of the classifier in correctly classifying instances even in the class imbalance conditions. The 0.9295 is considerably high, suggesting a strong correlation between predicted and true classifications. This indicates the robustness and reliability of the classifier's predictions. The high Kappa value of 0.9295 indicates substantial agreement beyond chance, reinforcing the reliability and consistency of the classifier's performance. The performance of LASSO with SVM (RBF) is due to the combined advantages of both methods; significant and distinct feature extraction method with for nonlinear pattern recognition and classification.

The second best results are reported from FPO-GMM Classifier with a high accuracy of 93.55% and F1 Score of 95%. This results also indicate a successful classification of microarray gene expression data with MCC and Kappa of 0.8591 suggesting a strong agreement between predicted and true classifications. If we compare with FPO-GMM and GMM, there is an improvement of 9.57% in accuracy, 7.8% in F1 Score, and 0.202 difference in MCC and Kappa. This is because FPO has truly contributed to optimizing the feature data of the Gaussian Mixture Model classifier. The FPO once again showed its ability to explore the solution space effectively. Overall, the LASSO with SVM (RBF) outperformed LASSO with FPO-GMM due to SVM's robustness in handling complex, nonlinear patterns inherent in microarray gene expression data. The performance analysis of classifiers with EHO feature extraction is next discussed through Table 10.

**Table 10:** Performance analysis of classifiers in colon cancer detection from EHO feature extraction.

S.No.	Classifiers	Accuracy	F1 score	MCC	Error rate	Kappa
1	GMM	90.32258065	92.30769231	0.794769584	9.677419355	0.792873051
2	PSO-GMM	91.93548387	93.67088608	0.82614923	8.064516129	0.825646794
3	DFA	88.70967742	91.13924051	0.756365507	11.29032258	0.755905512
4	NBC	90.32258065	92.30769231	0.794769584	9.677419355	0.792873051
5	Firefly-GMM	90.32258065	92.5	0.788636364	9.677419355	0.788636364
6	SVM (RBF)	95.16129032	96.20253165	0.895932952	4.838709677	0.895388076
7	FPO-GMM	96.77419355	97.5	0.929545455	3.225806452	0.929545455

**Figure 4:**
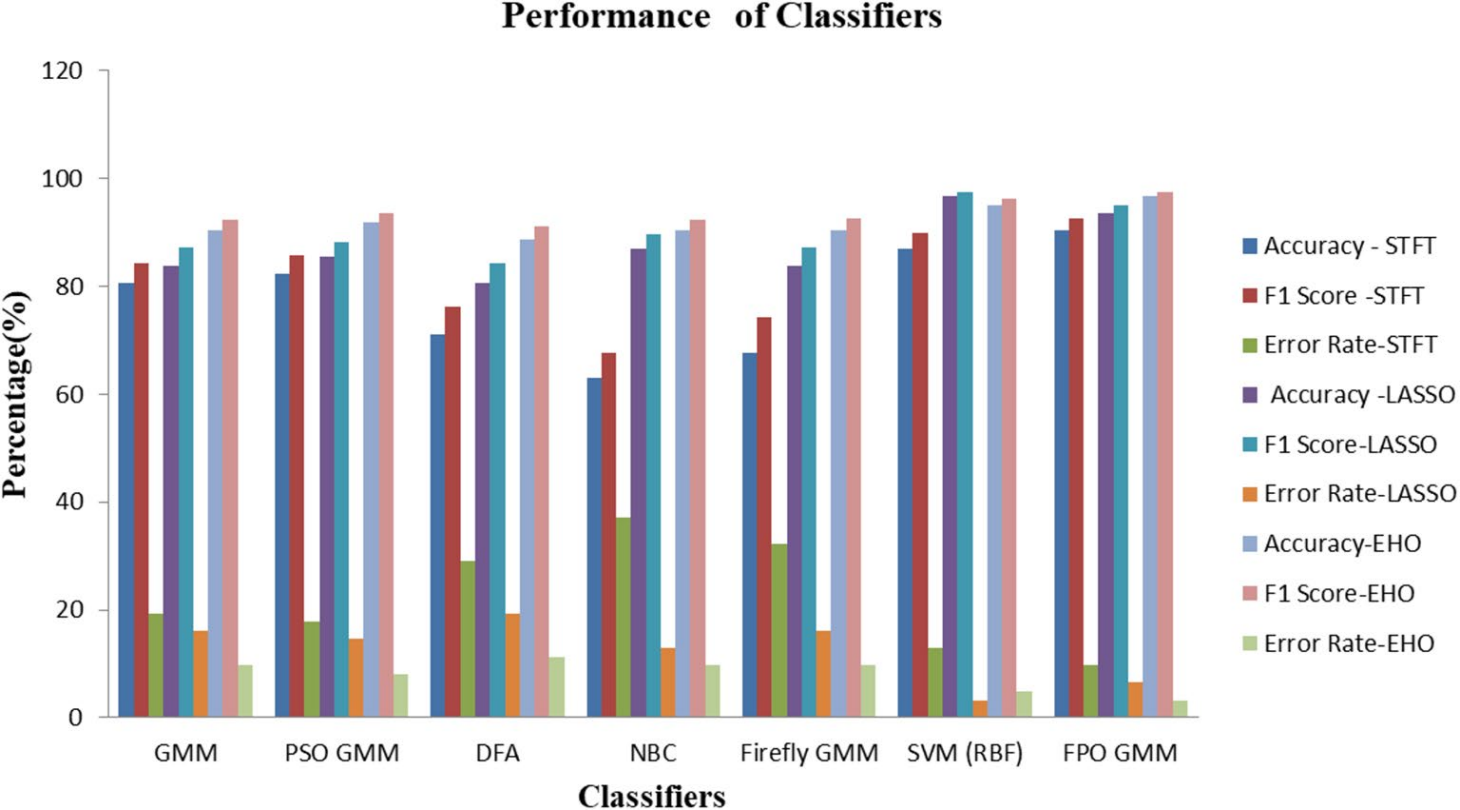
Employed classifiers with their classification performance metrics.

For the FPO-GMM classifier, a high accuracy of 96.77%, F1 Score of 97.5%, MCC of 0.9295, and Kappa of 0.9295 is reported for EHO feature extraction as provided in Table 10 values indicate successful classification of the data. The high F1 Score suggests effective handling of data imbalance, indicating balanced performance in capturing both positive and negative instances. EHO and FPO have contributed to optimizing parameters in both feature extraction and classifier tuning, respectively to enhance the model's performance. The second-best performance is observed with the SVM-RBF classifier. This is only a slight improvement of performance in in terms of accuracy of 1.61%, F1 Score of 1.3%, MCC of 0.03, and Kappa of 0.03. The similarity in MCC and Kappa values suggests that both classifiers achieved a similar balance between true positives, true negatives, false positives, and false negatives in the predictions. However, the difference in Accuracy and F1 Score is due to the variations in precision and recall values. The slight improvement in the performance of the FPO-GMM over the SVM-RBF to be attributed to the EHO feature extraction method that distinctly distributes the data in two clusters as provided in Figs. 2 and 3. In essence, STFT yields higher accuracy due to its ability to capture wide variability in expression levels across samples, potentially enabling better discrimination between classes. LASSO and EHO are normalized and sparse representations with more consistent expression levels across samples and hence they can distinguish even the subtle differences between classes. For a smooth comparison, Fig. 4 shows the performances of various employed classifiers with their classification performance metrics.

**Figure 5:**
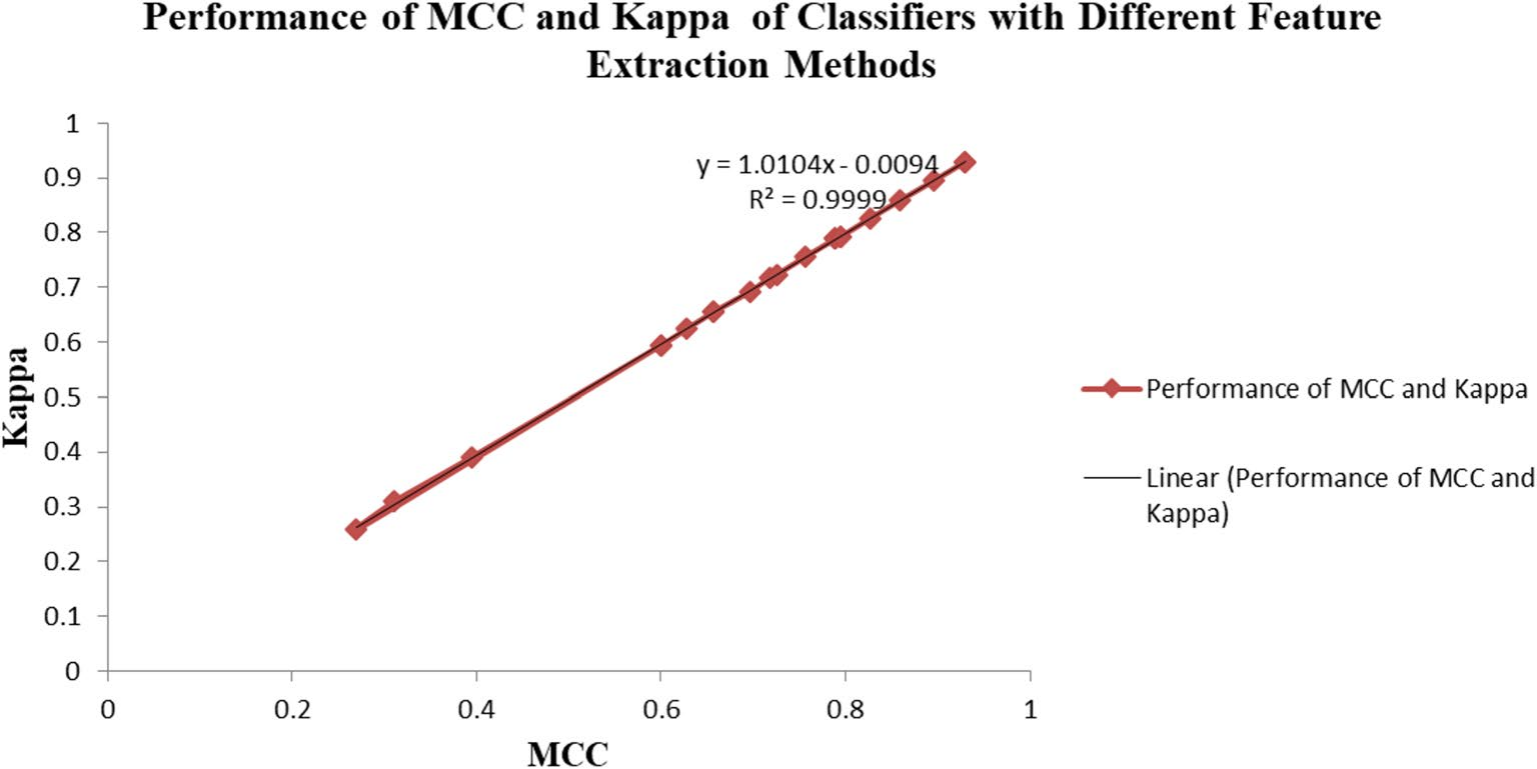
Performance of classifiers in terms of MCC vs. Kappa parameters for STFT, LASSO, and EHO feature extraction techniques.

Now, the overall performance of the various feature extraction methodologies and classification methodologies are compared using MCC vs. Kappa as portrayed in Fig. 5. The graph demonstrates a strong positive linear relationship between MCC and Kappa values for the various classifiers used with different feature extraction techniques. The high R^2^ value indicates that the relationship between MCC and Kappa values is highly linear and predictable. As one metric increases, the other tends to increase proportionally, suggesting that classifiers performing well in terms of Kappa also tend to perform well in terms of MCC. The linear relationship also suggests that the correlation between MCC and Kappa values are robust and not specific to any particular feature extraction method, especially for the imbalanced dataset in this research. Overall Fig. 5 is a representation of the reliable performance of the classifiers across various feature extraction techniques taken into consideration.

So, overall in the classification task, the choice between classifiers depends on requirements such as computational complexity, interpretability, or preference for certain optimization techniques. Overall, FPO-GMM and SVM (RBF) approaches offer effective solutions for classification tasks with microarray gene expression data, with the FPO-GMM approach providing a competitive alternative to traditional methods of SVM-RBF classifiers. In this upcoming section, the computational complexity comparison of the integrated feature selection and classification techniques are discussed.

### Computational complexity

The computational complexity of feature extraction and classification methods plays a crucial role in determining the feasibility and efficiency of the high volume of microarray gene expression data. In feature extraction, computational complexity refers to the time and memory resources required to transform raw data into a meaningful feature representation. The computational complexity in classification involves the resources needed to train and deploy classification models, as well as to make predictions on new data points. However, computational experience can vary across different hardware setups and systems. Therefore a robust understanding of the computational complexity of the utilized methods is comprehended with the Big O mathematical notation—O (n), with n indicating the size of the dataset. The computational complexity O (n) indicates that the computational effort grows linearly with the dataset size ‘n’. Table 11 provides the computational Complexity of various employed methodologies for Classification.

**Table 11:** Computational complexity of various employed methodologies for classification.

Classifiers	Without feature extraction	With STFT feature extraction	With LASSO feature extraction	With EHO feature extraction
GMM	O(2n log2n)	O(2n^4^ log2n)	O(2n^3^ log2n)	O(2n^4^ log2n)
PSO GMM	O(2n^3^ log2n)	O(2n^7^ log2n)	O(2n^4^ log2n)	O(2n^5^ log2n)
DFA	O(n)	O(2n^3^)	O(n^3^)	O(n^4^)
NBC	O(n log2n)	O(2n^3^ log2n)	O(n^3^ log2n)	O(n^4^ log2n)
Firefly GMM	O(2n^2^ log2n)	O(2n^4^ log2n)	O(2n^4^ log2n)	O(2n^4^ log2n)
SVM (RBF)	O(n log n)	O(2n^2^log n)	O(n^3^ log n)	O(n^4^ log n)
FPO-GMM	O(2n^5^ log2n)	O(2n^7^ log2n)	O(2n^7^ log2n)	O(2n^8^ log2n)

Under the various feature extraction techniques, the EHO feature extraction with the FPO GMM classifier has the highest complexity of O (2n^8^ log2n). The lowest computational complexity of O (n^4^) is observed in DFA classification. From Table 8, 9 and 10 shows that the methodology with comparatively with high computational complexity has provided better results during classification. When dealing with high-dimensional data, like microarray gene expressions, feature extraction helps to reduce the dimensionality and extract relevant information, making the subsequent classification task more manageable for complex classifiers. Also during classification, to bring out the underlying patterns and nonlinear relationship of the data, often the classifiers employed need to be more complex as the gene expression data is not easily separable in the original feature space.

Overall, feature extraction followed by classification methods with high computational complexity is preferred in situations where achieving optimal performance, handling complex data patterns, enhancing robustness to noise, and scalability are essential considerations. So when computational resources are available, these complex learning models can be used to classify microarray gene expression data. Likewise, feature extraction followed by classification methods with lower computational complexity are preferred, for real-time applications where rapid decision-making within the time and resource limit is critical.

Here is the gist of the research and adopted methodology employed in the paper. We begin by outlining the feature extraction methods employed, namely STFT (Short Term Fourier Transform), LASSO (Least Absolute Shrinkage and Selection Operator), and EHO (Elephant Herding Optimisation). We explained how these methods were used to reduce the dimensionality of the microarray gene expression data and extract meaningful features for the classification task. After that, we discussed the integrated machine learning and metaheuristic classification techniques utilized in this study. Then we provided insight into the classifiers employed, including the Gaussian Mixture Model (GMM), Particle Swarm Optimization (PSO) with GMM, Detrended Fluctuation Analysis (DFA), Naive Bayes classifier (NBC), Support Vector Machine with Radial Basis Kernel (SVM-RBF), and Flower Pollination Optimization (FPO) with GMM. After that, we elucidated how these classification techniques were applied to accurately classify colon cancer based on the extracted features. Following this, we presented the results obtained from the evaluation of our model. The performance of the classifiers was evaluated using the metrics: Accuracy, F1 score, Matthews correlation coefficient (MCC), and Kappa statistics. We also highlighted the significance of utilizing STFT, LASSO, and EHO for feature extraction in reducing the dimensionality of microarray gene expression data. The results compared with existing benchmark studies provided in Table 1 emphasize the novelty and effectiveness of our approach in enhancing classification accuracy and interpretability for colon cancer detection. The conclusions, limitations of research, and future research directions are discussed in the next section.

## Conclusion

This research contributes to the ongoing fight against colon cancer by exploring novel methods for early detection and improved diagnosis. The major focus of the research is to address the curse of the dimensionality problem inherent in microarray data and to extract significant features that retain relevant patterns and intrinsic nonlinearity. An accuracy of 96.77% is reported for LASSO feature extraction with SVM-RBF Classification. Likewise, 96.77% is also reported for EHO feature extraction with FPO-GMM Classification. This indicates the balance between feature extraction and classification of both approaches employing different optimization techniques. Both methodologies worked well to discover the unseen data, indicating that the selected features are relevant. From the computational complexity analysis, it is observed that LASSO feature extraction with SVM-RBF Classification will incur less computational complexity when compared to EHO feature extraction with FPO-GMM Classification. So the choice of methodology can be made based on computational complexity, suitability, and efficiency. However, the effectiveness of metaheuristic optimization techniques, such as PSO and FPO may vary across different datasets and problem domains. The metaheuristic optimization methods are computationally intensive and often require tuning of algorithm parameters using hyperparameter tuning to further optimize and reproduce the same research results. So the future direction of the research is to utilize techniques like grid and random search to systematically explore the hyperparameter space and identify optimal settings for metaheuristic and machine learning classifiers. Also t-SNE, ReliefF methods for dimensionality reduction can be experimented to discriminate the high-dimensional data and identify underlying patterns and clusters.

## Data Availability

The datasets used and analysed during the current study are available from the corresponding author on reasonable request.

## List of symbols

$\varphi$: Regression coefficient in LASSO
$\lambda$: Slope coefficient in LASSO
$\eta$: Control variable in EHO
$\upxi$: Control variable for Global best in EHO
$\text{M}$: Coefficient in GMM
$\Upsilon$: Mean vector in GMM
$\text{C}\cdot$: Covariance matrix in GMM
$\alpha$: Randomization parameter in FA
$\beta$: Attraction coefficient in FA
$\Upsilon$: Light absorption coefficient in FA
$\sigma$: Kernel width parameter in SVM
$\delta$: Step size in FPO
$\text{L}$: Levy flight step size for small step size in FPO
$\Gamma$: Levy flight step size for large step size in FPO
$\varepsilon$: Step size for the random walk in FPO
